# Tool Wear Issues in Hot Forging of Steel

**DOI:** 10.3390/ma16020471

**Published:** 2023-01-04

**Authors:** Janusz Krawczyk, Aneta Łukaszek-Sołek, Tomasz Śleboda, Łukasz Lisiecki, Michał Bembenek, Jacek Cieślik, Tomasz Góral, Jan Pawlik

**Affiliations:** 1 Faculty of Metals Engineering and Industrial Computer Science, AGH University of Science and Technology, Av. Mickiewicza 30, 30-059 Krakow, Poland; 2 Faculty of Mechanical Engineering and Robotics, AGH University of Science and Technology, Av. Mickiewicza 30, 30-059 Krakow, Poland

**Keywords:** hot forging, steel, microstructure, wear mechanisms

## Abstract

Steel forging tools are subjected to a number of tribological wear mechanisms depending on the geometry and surface of the tool and the flow of the material. Thus, there is no single general tribological wear mechanism, and only the predominant wear mechanisms in this case can be indicated. The problem has been known for years, but due to its complexity research on it is still relevant. In this study, the various wear mechanisms of hot work tools are analyzed on the basis of original research. Moreover, the influence of the micro- and macrostructure of the material and of its mechanical, physical, and technological characteristics on susceptibility to a given type of wear is considered. Adhesive wear, wear caused by plastic deformation, mechanical fatigue, thermal fatigue, the influence of hardness, heat treatment, and impact strength on tool wear and the mechanisms causing this wear are discussed in addition to tribological wear mechanisms such as abrasive wear. The influence of thermomechanical history and the characteristics of the tool material, including structural anisotropy, on the wear of these tools is indicated. The analysis of wear mechanisms performed will enable more precise definition of the principles of tool material selection and tool material condition for the hot forging of steel.

## 1. Introduction

Tribological interaction can significantly change the microstructure in the surface layer of tools. The mechanical interaction is the cause of plastic deformation, which results in changes in the microstructure occurring in the superficial layer. The plastic deformation of the surface layer is associated with its strengthening [1,2,3,4,5,6,7,8]. However, it should be noted that in the case of adhesive wear [8] or the tempering influence of heat released during friction [9], the hardness of the surface layer, despite plastic deformation, may be lower than that of the rest of the tool material. Proper characterization of the mechanism of formation and of the tribological properties of the surface layer therefore requires determining the difference in its mechanical properties compared to the rest of the tool material. In particular, theories linking the changes in mechanical properties as a result of plastic deformation to the nucleation of cracks in the surface layer require a description of the properties of the surface layer. In some cases, these changes can lead to catastrophic wear [10,11,12]. Many studies confirm the important role of plastic deformation of the surface layer in the formation of cracks on the surface of iron alloys that have been subjected to tribological interaction [13,14,15], including hot forging dies, for instance [16]. Tribological interaction results in a change in the state of stress in the surface layer. These changes can result from mechanical interaction alone, but also (very often simultaneously) from thermal interaction. A common result of such interactions is the formation and development of cracks. Most often, such cracks are of the so-called fatigue nature. This is due to the fact that the operating conditions of the tribological systems in various devices are characterized by varying loads. The development of fatigue cracks is often analyzed on the basis of their geometry and explained only by the state of stress without considering the microstructure [17,18,19,20,21,22,23,24,25,26]. However, the fracture toughness of the materials is closely related to their microstructure [27,28,29]. Thus, the path of crack growth may also depend on the morphology of the phases [30,31,32,33,34,35]. For single-phase materials, grain boundaries may play an important role in crack development [36,37,38]. Similar relationships between cracking and microstructure can occur in fatigue wear [39,40,41,42,43,44]. However, often when conducting observations or calculations to predict the morphology of thermal fatigue wear crack mesh, for simplicity’s sake, a complex factor related to the morphology of precipitates, such as carbides, is not taken into account [45,46,47,48,49,50]. This is due to the complexity of this issue as well as to the problems of controlling each component (tool) in terms of microstructure when optimizing its operating conditions. In the case of complex shapes of the working surface of a tool, depending on its surface area, there is a differentiated impact of the material being processed on this surface [51]. This can result in variation in the main wear mechanisms, despite the fact that the tool was made of the same, seemingly uniform material. Such a conclusion was made in one study [52] on the basis of analysis of the wear mechanism of a forging die. It is often observed that cracks are initiated in areas where there is a pronounced change in the curvature of the tool surface [53,54,55]. This is due to the concentration of stresses in the area where notches occur on the surface of the workpieces. On the other hand, an important microstructural factor that facilitates such cracking is the presence of carbides [56] and especially their precipitates arranged in bands [57]. The working conditions of the die depend strongly on the area of the die cavity surface. The test methodology used includes technological tests, i.e., [58,59,60,61,62], unlike many studies based on laboratory tests, which give greater control of testing parameters but only deal with approximate operating conditions [63,64,65,66].

In this study, various wear mechanisms in hot working tools are analyzed on the basis of original research. In addition, the influence of the micro- and macrostructure of the material and its mechanical, physical, and technological characteristics on susceptibility to a given type of wear is introduced for consideration. Adhesive wear, wear caused by plastic deformation, mechanical fatigue, thermal fatigue, the influence of hardness, heat treatment, and impact strength on tool wear and the mechanisms causing this wear were analyzed in addition to tribological wear mechanisms such as abrasive wear. The role of history and tool material characteristics, including structural anisotropy on the wear of tools, was indicated. The analysis of wear mechanisms carried out will enable a more precise determination of the principles of hot forging tools material selection and of the condition of the material.

## 2. Materials and Methods

**Figure 1:**
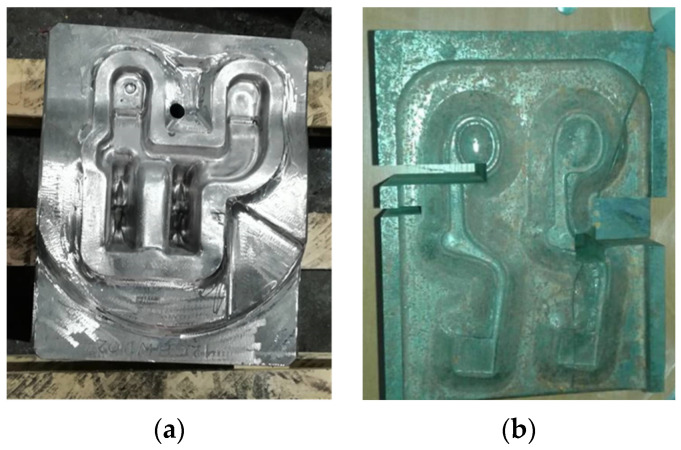
The investigated hot forging tools: (**a**) insert for forging “fork-type” forgings, (**b**) forging die designed for forging wound spreader components.

Hot forging tools (Figure 1) made out of hot working tool steels were analyzed. Representative examples were selected to identify a range of tribological wear mechanisms depending on the geometry of the tool surface relative to the plastic flow of the material.

An analysis of the various mechanisms of hot working tool wear was carried out on the basis of tests of the bottom insert of a finishing die made of X40CrMoV5-1 steel, which occurred during the forging of a “fork-type” part on an LKM 2500 press (Smeral, Czech Republic) (Figure 1a) as well as on the basis of tests of a die made of 55NiCrMoV7 tool steel used for the hot forging of martensitic steel wound spreader on an MPM 1600 hammer (Huta Zygmunt Steel Mill, Poland) (Figure 1b). In both cases, the tools were taken out of service due to their destruction. The chemical composition of the investigated tool steels is presented in Table 1.

**Figure 2:**
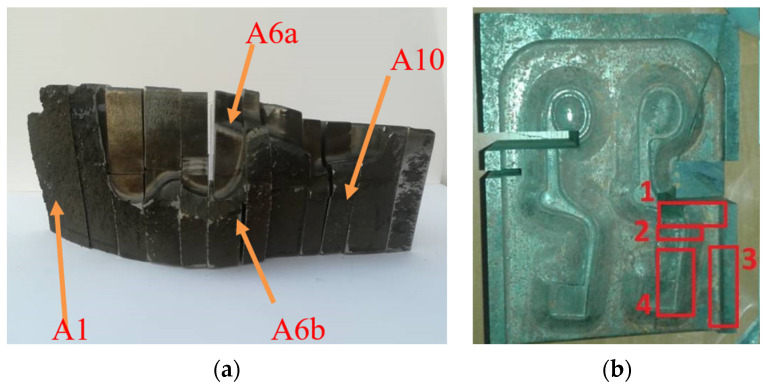
The method of taking samples for testing: (**a**) a fragment of an insert for forging “fork-type” forgings; (**b**) the die for forging wound spreader components.

The tools were analyzed using light microscopy and scanning microscopy, paying special attention to the study of fracture and microstructure. The hardness of the insert for forging “fork-type” parts was measured using a Brinell hardness tester with a load of 3000 kG (the diameters of the imprints were converted to HB hardness in accordance with a PN-EN 6506 standard) and hardness measurements using a Rockwell hardness tester. The microstructure of the tested forging insert was observed on an Olympus PMG3 microscope (Tokyo, Japan) and a Carl Zeiss AxioVert 200 MAT light microscope (Oberkochen, Germany). The examined metallographic cross-sections were etched with 5% and 2% nital. Observations of the fracture surface were made using a scanning electron microscope (Japan Electron Optics Laboratory Co., Ltd., Tokyo, Japan). Material was taken from the damaged tools for impact testing on a 15 kN Charpy hammer on specimens with a 2 mm–deep V-shaped notches. Figure 2 shows how the samples were taken for testing. In the case of the forging die for forging a “fork-type” part (Figure 2a), test specimen locations were selected and marked to clarify the causes of cracking. The sample marked A1 was used to test the chemical composition to confirm the grade of the material from which the die was made; the sample A6a was subjected to macroscopic examination of the near-surface layer using fractographic observations (SEM); the sample marked A6b was used for microscopic examination of the near-surface layer, macrostructure (banding), and microstructure (light microscope); and the sample marked A10 was used to measure hardness.

The samples taken from the die shown in Figure 2b were respectively labeled: 1—for banding and microstructure studies in three (X, Y, Z) planes; 2—for wear mechanism and hardness studies in the zone near the die working surface; 3—for impact testing; and 4—for die working surface studies using SEM.

## 3. Results and Discussion

### 3.1. Die Insert for Forging “Fork-Type” Forgings

#### 3.1.1. Macroscopic Evaluation of Die Damage

**Figure 3:**
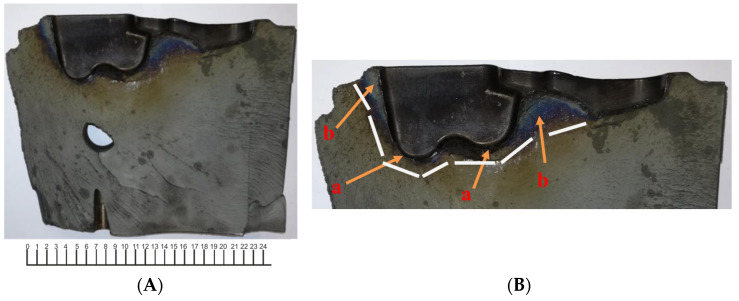

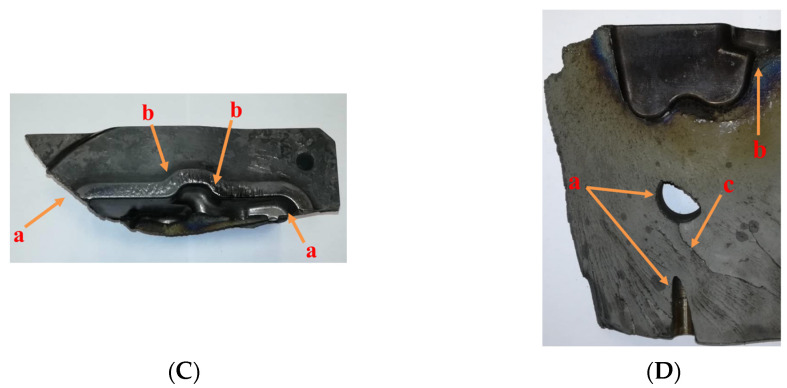
Cracked die insert for forging “fork-type” forgings: (**A**) view of the fracture surface, (**B**) the fracture with the deepest areas of the die insert cavity (marked “a”) and the extent of heat influence deep into the die insert (blue fracture surface, marked “b”); white line indicates the range of fracture surface oxidation during tool operation before its destruction, (**C**) with the locations of the intersection of the crack with the flash (marked “a”) and the wear marks on the flash bridge (marked “b”), (**D**) with the location of the holes for fixing the forging dies (marked “a”), the area of fatigue cracking (marked “b”), and the area of material “final fracture” (marked “c”).

Macroscopic analysis of the die insert was performed to evaluate crack development (Figure 3a). Crack propagation took place along the deepest areas of the die insert cavity. These spots are characterized by a variable geometry reflecting the geometry of the lower part of the forging. The blue color of the fracture area near the die working surface is also characteristic; it testifies to the extent of the influence of the heat transferred by the forging semi-products heated to 1200 °C on the oxidation conditions of the fracture surface (Figure 3b). It can be noticed that the temperature in the abovementioned fracture area was about 400 °C. Another factor that affected the destruction of the die was the pressure exerted on the flash bridge during the forging. The excessive pressure of the material being formed caused high stresses to build up at the bridge–die cavity interface. The forging of only 80 pieces resulted in tribological wear of the bridge (as shown in Figure 3c) that was disproportionately high to the range of operation. Such wear should be characteristic of a die insert on which about several hundred pieces of such a workpiece were forged. Other factors that affected the durability of the die insert are die fixing holes. They were placed several centimeters below the deepest points of the die cavity and weakened the structure of the die insert, thus contributing to its faster destruction. Figure 3d shows lines, the outline of which runs around and through the die fixing holes, which indicates that the crack propagating in this area was already within the range of the so-called “final fracture” and therefore outside the range of fatigue cracking.

#### 3.1.2. Evaluation of Macrostructural Banding of Damaged Die Insert

**Figure 4:**
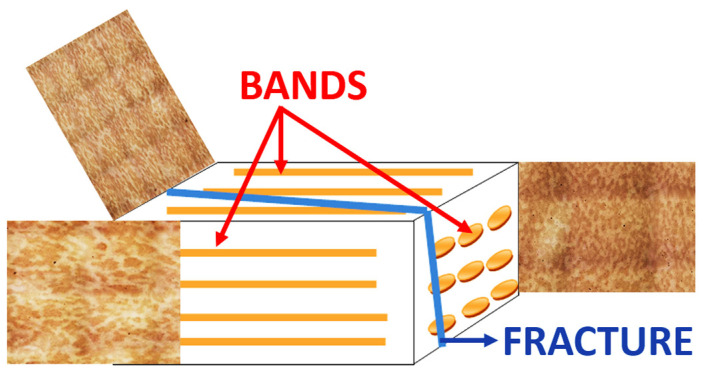
Image of the etched surface of die insert cross-section.

Crack propagation took place along the deepest parts of the die cavity. The material was found to be pre-formed to a small degree. The dendritic structure is still visible (Figure 4). This indicates a very low degree of forging of the ingot used to make the dies. This is a manufacturing error made at the material preparation stage, which is not correctable after machining of the die cavity and heat treatment. The weak banding is oriented parallel to the split plane of the die insert, and its direction is consistent with the direction of the longer side of the tool. The resulting fracture is related to the banding structure, formed along the plane of the occurrence of the bands (Figure 4). This is a characteristic area of fatigue crack generation in forging dies (die working surface), primarily in the vertical plane and perpendicular plane to the direction of movement of the press crosshead.

#### 3.1.3. Evaluation of the Microstructure of a Damaged Die Insert

**Figure 5:**
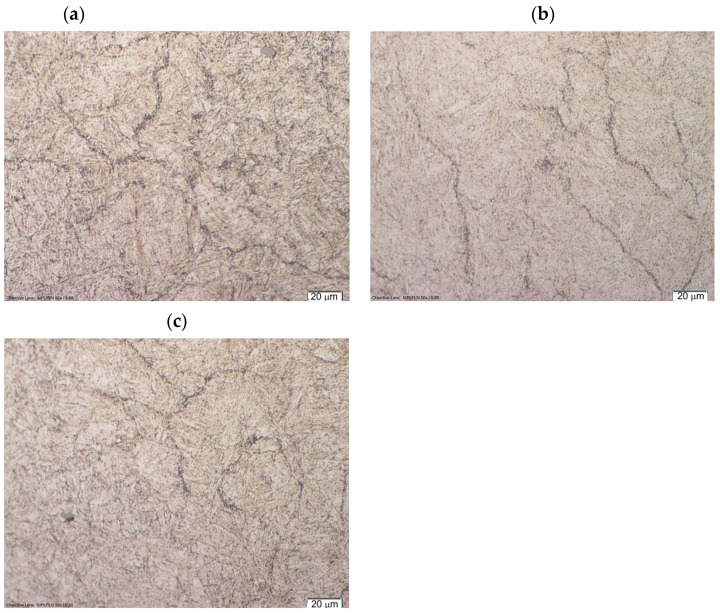
Microstructure of die insert (sample A6b) observed at: (**a**) XY plane, (**b**) ZX plane, (**c**) ZY plane.

The grain boundaries of former austenite are clearly visible in the microstructure of the die insert (Figure 5). This indicates the precipitation of carbides at these boundaries. These precipitates will facilitate crack propagation and may be formed during quenching as secondary carbides or during tempering as transitional carbides. In addition, conglomerates of nonmetallic inclusions can be seen in the bright interdendritic areas. The observed non-metallic inclusions are sulfides, oxides, and nitrides. The aforementioned nonmetallic inclusions may be a factor facilitating cracking.

**Figure 6:**
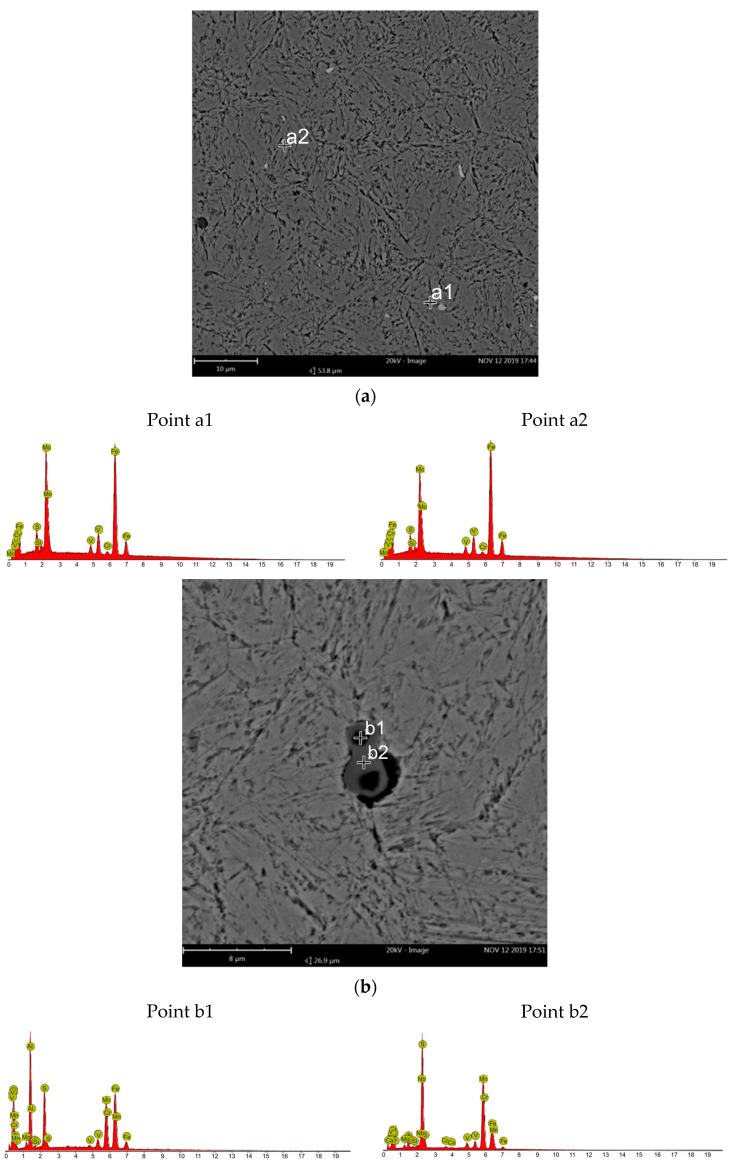
Microstructure of die insert alloy matrix material with results of microanalysis of the che-mical composition (EDS) of microstructure components (SEM) at two different areas of the die insert (**a**,**b**).

Images of the microstructure obtained with a scanning electron microscope indicate a die insert alloy matrix being a tempered martensite. Bright carbide precipitates and dark nonmetallic inclusions are evident. At the grain boundaries of the former austenite, dispersive particle precipitations are visible. In order to identify carbide precipitates and non-metallic inclusions, microanalysis was performed in their areas using a scanning electron microscope (Figure 6). The microanalysis performed in the area of the bright precipitates indicates molybdenum carbides (probably primary carbides). The gray precipitates are manganese sulfides and the dark precipitates are aluminum oxides.

#### 3.1.4. Surface Layer in the Tribological Contact Area

**Figure 7:**
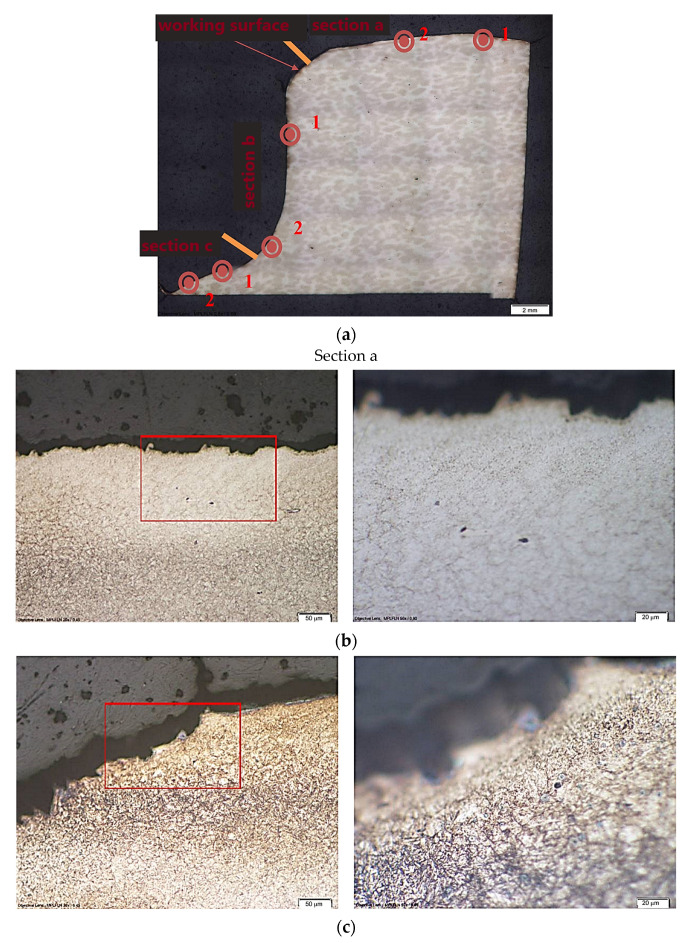

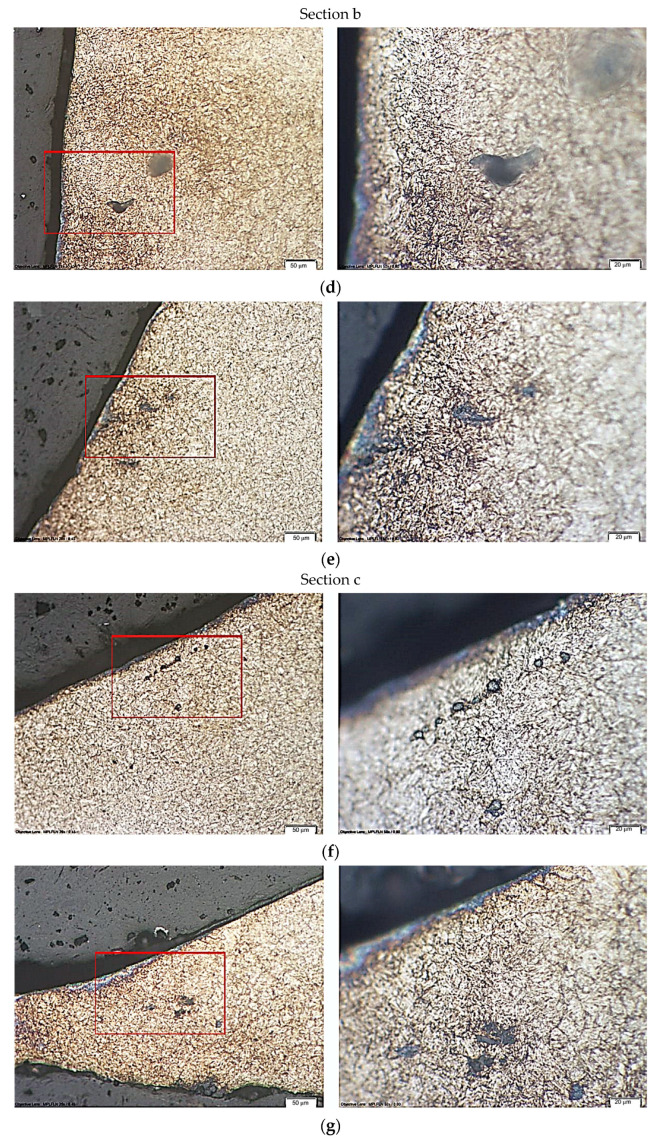
Microstructure of the investigated die insert in the near-surface layer with image magnification (area marked with red box): (**a**) areas selected for the analysis, (**b**) observation area 1 section a, (**c**) observation area 2 section a, (**d**) observation area 1 section b, (**e**) observation area 2 section b, (**f**) observation area 1 section c, (**g**) observation area 2 section c.

The microstructure of the tool’s near-surface layer was analyzed to determine the effect of tribological wear. The microstructure was analyzed at the locations marked in Figure 7. At section “a”, wear occurs through chipping of the tool material (Figure 7b). The microcracks appear to be correlated with the boundaries of the former austenite grain (Figure 7c). On section “b”, the dominant wear mechanism is probably abrasive wear (Figure 7d,e). No clear tribological wear mechanism is observed in section c, and it is an area mainly exposed to high-temperature oxidation.

#### 3.1.5. Fractography of the Die Insert Fracture Surface

**Figure 8:**
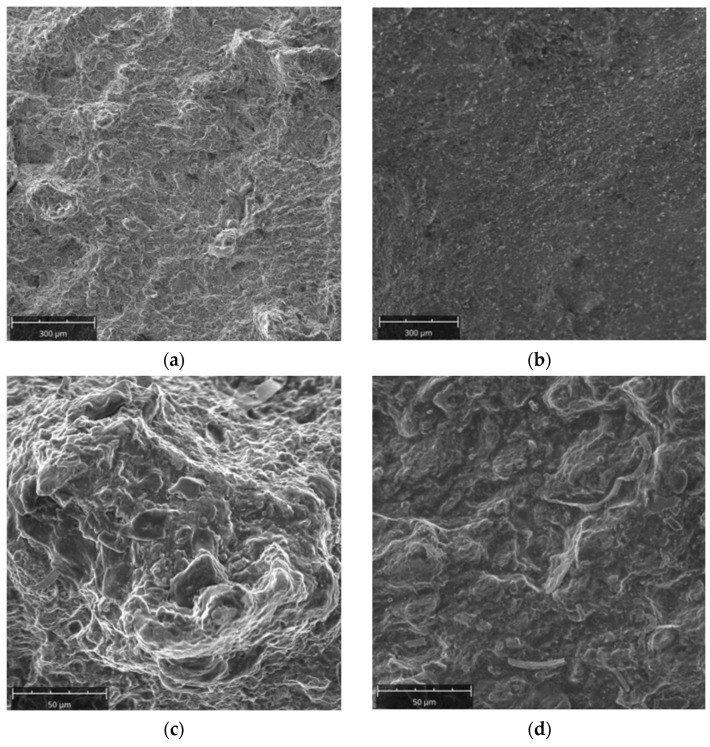
Oxidized fracture surface of the investigated die insert: (**a**) visible fatigue striations, (**b**) dominance of transcrystalline fracture, (**c**,**d**) intercrystalline fracture areas.

The die insert fracture surface was analyzed using scanning electron microscopy. Sample A6a was selected for testing. Despite the oxidation of the fracture surface, the surface’s very brittle character with intergranular and transcrystalline fracture can be observed (Figure 8).

#### 3.1.6. Hardness and Fracture Strength Testing

Hardness measurements were performed on the side surface and on the bridge of the tool. A hardness of 52 HRC was measured, which is higher than the design assumptions. Hardness was also measured by the Rockwell method (without using conversion) on the cutting surface at distances of 2.5, 3, and 4 mm from the working surface of the die cavity. The results of the hardness measurements (43 HRC, 45 HRC, 50 HRC), obtained in order, indicate that it decreases toward the die working surface. The effects of the thermal influence of the material being processed on the tool cause a decrease in its hardness, and it obtains the hardness which is in agreement with the technical standard at a depth of about 30 mm below the die working surface. Analyzing the results, it should be concluded that during the heat treatment the die was tempered to a secondary hardness peak, that is, at a temperature of about 450–500 °C, instead of using tempering at higher temperature, i.e., at a temperature of about 650 °C. Tempering to a peak of secondary hardness is directly related to the decrease in impact strength of the material. On the other hand, as a result of the loss of hardness associated with the increase in tempering during contact with the charge material heated to 1200 °C, the initial hardness did not play a role in the improvement in tribological properties and even resulted in their reduction, which was already observed during the die exploitation.

**Figure 9:**
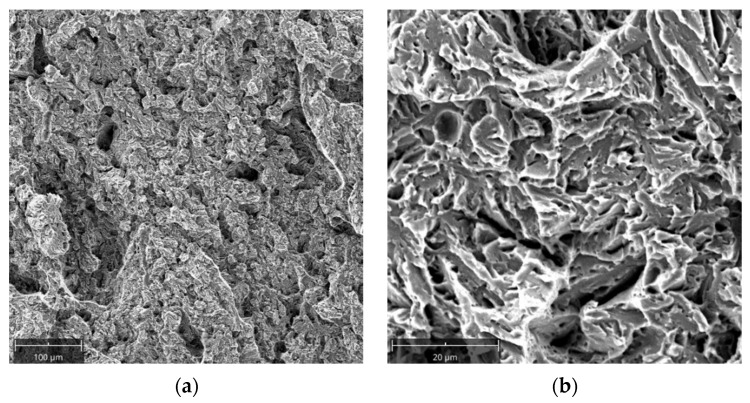
Fracture surfaces of the investigated die insert: (**a**) low magnification, (**b**) high magnification.

Samples for Charpy testing were cut perpendicular to the fracture surface of the tested die insert. The obtained results (4.6 J, 3.5 J, 3.8 J) indicate that the material was very brittle. The increased hardness of the insert and the presence of carbides at grain boundaries drastically reduced the impact strength. Observations were made on a scanning electron microscope to determine the type of fracture characteristic of the tested die insert. It can be observed that the fracture proceeds along the so-called planes of splitting (Figure 9). Intergranular fracture is also visible. Moreover, decohesion most likely along areas of intense etching of the microstructure, i.e., places of probable separation of transition carbides, can also be noticed. The areas indicating the fracture along the interphase boundaries (nonmetallic inclusion/metallic matrix) can also be observed.

### 3.2. Forging Die for Forging Wound Spreader Components

#### 3.2.1. Macroscopic Evaluation of the Damage in Terms of Banding

**Figure 10:**
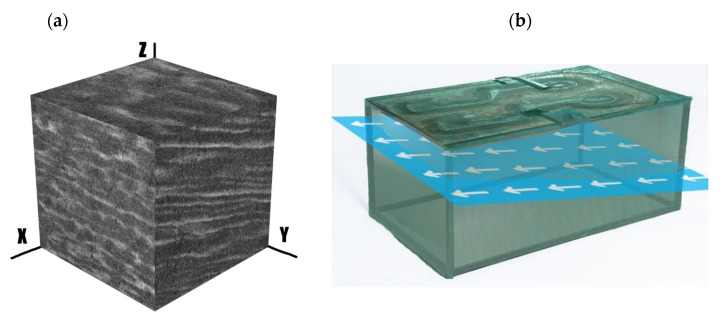
Banding in relation to the forging die geometry: (**a**) 3D image of banding structure, (**b**) alignment of bands in relation to forging die geometry.

The die alloy is characterized by a highly banded structure (Figure 10a). Figure 10b shows the arrangement of the bands relative to the geometry of the die. The arrangement of the bands was appropriate to hinder crack initiation, but further analysis of the geometry of the die fracture showed the important role of the banding for its development. It was found that the die cavity was made on the correct die surface, but the edges of the die cavity should be aligned perpendicular to the band’s alignment. The die fracture, which ended its service life, was due to improper geometric alignment of the die cavity relative to the geometry of the bands.

#### 3.2.2. Microstructure of Forging Die

**Figure 11:**
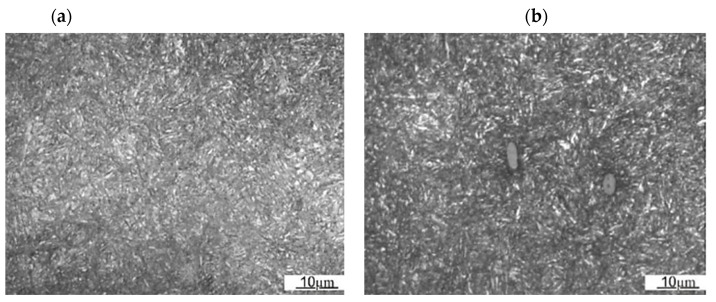
Microstructure of the tested forging die material: (**a**) area between bands, (**b**) area inside bands.

The microstructure of a forging die consists of tempered martensite. One can observe the local occurrence of tempered lower bainite in areas outside the bands (Figure 11a). This indicates the lower hardenability of the tested material in the center areas of the former dendrites. In the band areas (originally interdendritic areas), the martensite needles are finer. This indicates that there are some differences in the material’s properties inside and outside the band areas. Nonmetallic inclusions in the form of sulfides were also observed (Figure 11b), which correspond to the original interdendritic areas. Nonmetallic inclusions and, more precisely, the boundaries between them and the tool alloy matrix, can be a factor in the development of fracture and can even be sites of crack nucleation. However, the contribution of sulfides should be considered acceptable especially with regard to the fracture mechanics of hard tool steels.

#### 3.2.3. Morphology of the Forging Die Surface Layer

**Figure 12:**
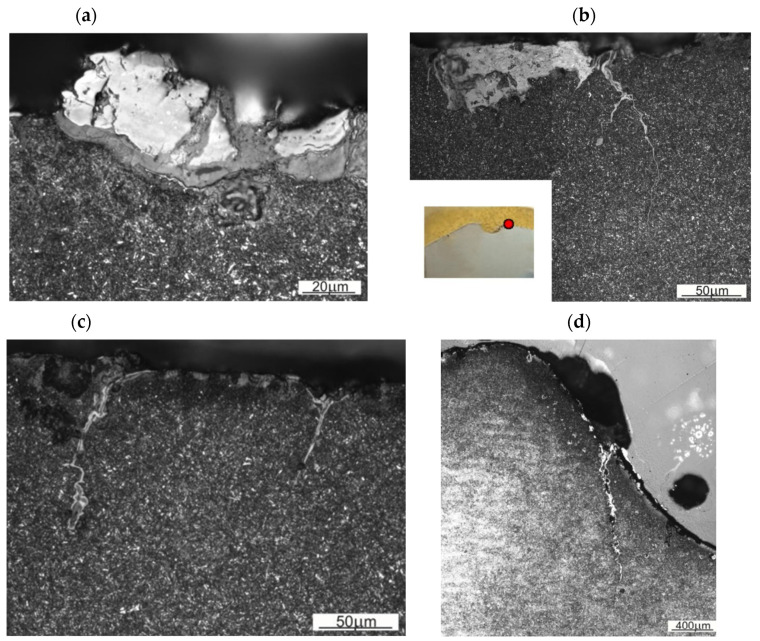
Changes in the die surface layer: (**a**) multilayer oxidation products, (**b**) the effect of fatigue-corrosion crack initiation near the area of intense oxidation of the surface layer of finishing die cavity (shown in **b**—die cavity on the right side; red dot indicates the area of metallographic observations.) for forging wound spreader components, (**c**) microcracks that are the nucleus of further fatigue-corrosion cracking, (**d**) a crack in the area of the edge of the die cavity from the center of the die.

One of the wear mechanisms of the surface layer is oxidation, which proceeds without a clear effect of microstructure on its development. The multilayer nature of oxidation products is evident (Figure 12a). Notches can form in the areas of oxidation-induced material loss, which facilitate the further development of fatigue-corrosion cracks (Figure 12b). Such cracks are characteristic of the surface layer of the tested forging die. In this case, changes in the surface layer caused by the processes of tempering the die material and plastic deformation are often visible (Figure 12c). Plastic deformation of the tempered surface layer results in plastic flow of the die material, resulting in microcracks that are the nucleus of further fatigue-corrosion cracking. Places particularly exposed to abrasive wear include the edges of the die cavity. In the areas of the edges of the die cavity, there are areas particularly exposed to the formation of deep fatigue-corrosion cracks. Figure 12d shows a microstructure where the fatigue-corrosion cracks (or several of them) in the area of the die cavity edge developed into a fracture that eventually led to die failure.

**Figure 13:**
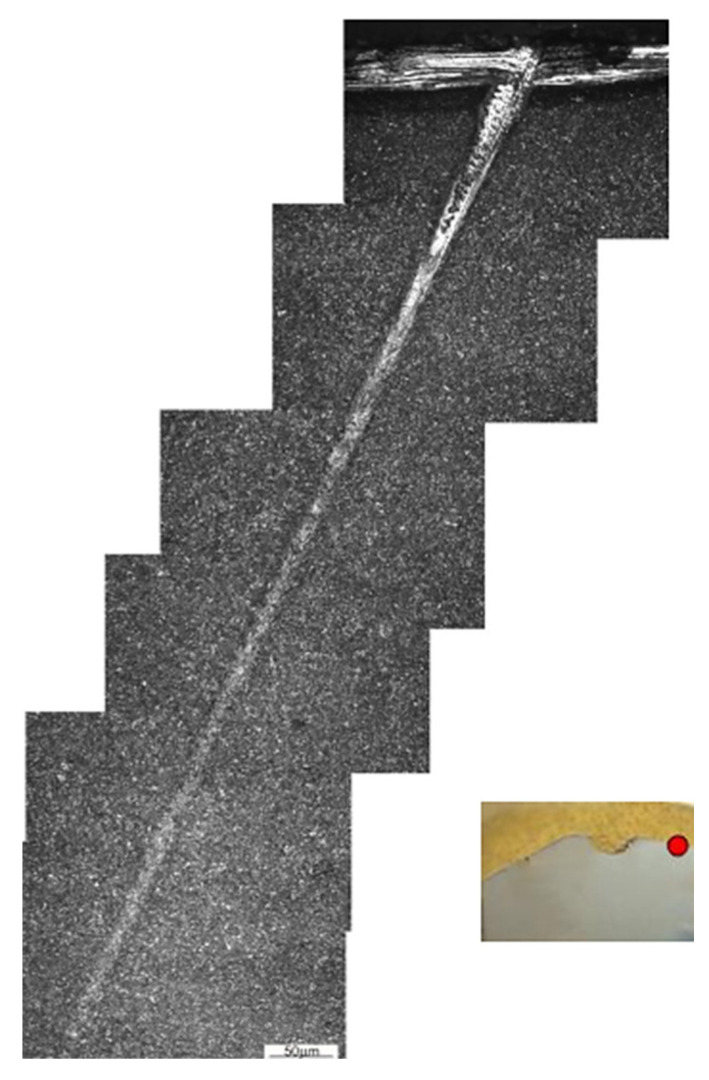
Effect of changes in die material microstructure due to intense subsurface interaction (adiabatic shear band). Red dot indicates the area of metallographic observations.

An adiabatic shear band was found in the near-surface zone of the tested die (Figure 13). The formation of the adiabatic shear band should be associated with the poor operation of the die, connected with striking the upper and lower dies without charge material. Shear stresses accumulate at the point of tools contact, resulting in the formation of a shear band. In the area of this shear band, concentrated plastic deformation leads to an increase in the temperature above the austenitic transformation temperature, further plastic deformation of the austenitic structure under adiabatic conditions (i.e., without heat exchange), and then rapid cooling, resulting in a very hard and brittle structure developing deep into the die material. Further operation of such a die can result in crack initiation along the adiabatic shear band and the formation of macroscopic damage in the die.

#### 3.2.4. Analysis of Forging Die Surface

**Figure 14:**
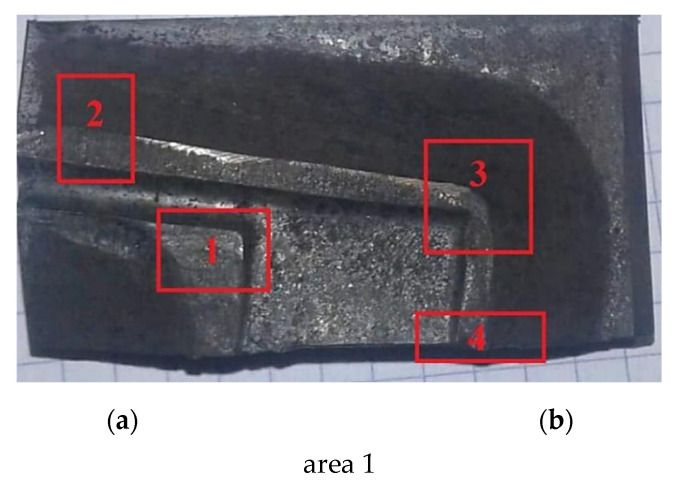

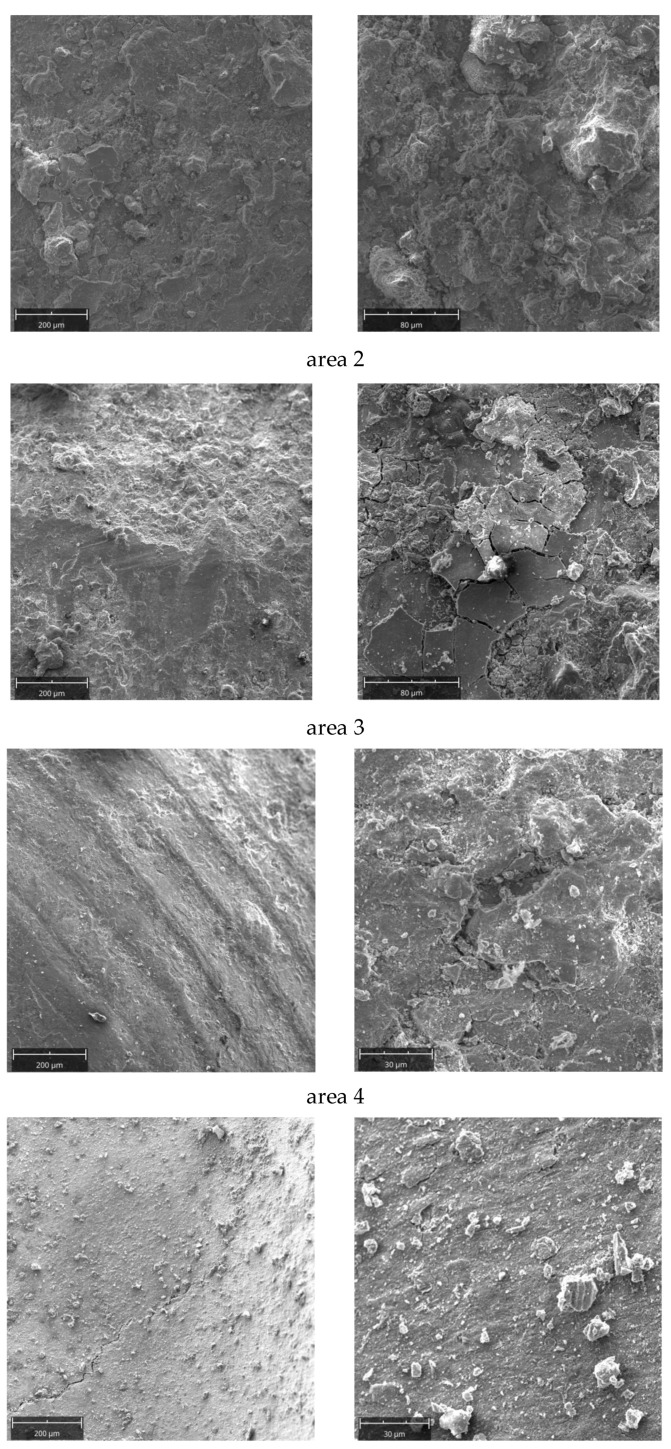
A fragment of the die selected to study its surface. Four areas (**1**–**4**) analyzed are marked: (**a**) low magnification, (**b**) high magnification.

For the analysis of the surface of the matrix, a fragment corresponding to the forming of the arm was selected at the point of its transition to the blade area, from which the teeth will be cut in the subsequent stages of production of the abovementioned wound spreader. This fragment with marked areas subjected to electron microscopic observation is shown in Figure 14. Examination of area 1 reveals a significant contribution to the wear of the die by its oxidation. In area 2, in addition to wear by oxidation, the effects of abrasive wear on the die are also apparent. Oxidation and chipping of the oxide layer is also characteristic of area 3. In this case, remnants of die machining can also be observed in the area outside the contact of the shaped forging. The grooves remaining after surface machining indicate that the crack initiation may be facilitated by leaving them on the surface after die cavity machining. Inside these grooves, fatigue-corrosion crack nucleation will be facilitated. Analysis of the area 4, located near the resulting crack, indicates erosive wear of the surface layer with visible oxidation products.

#### 3.2.5. Analysis of Fracture Propagation

**Figure 15:**
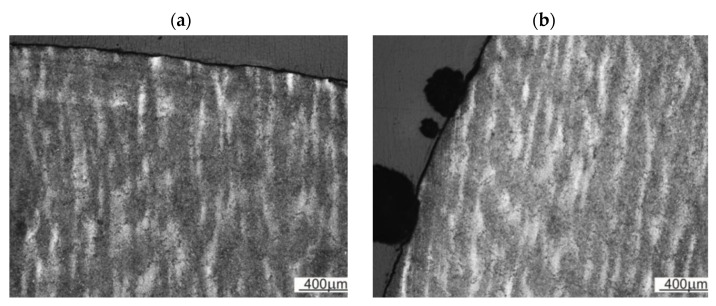
Path of fracture development in relation to the banding structure of the die.

The study of crack development made it possible to present the change in the path of crack development in the widest possible range. The crack at the initial stage proceeded along the direction perpendicular to the die surface (perpendicular to the bands—Figure 15a) and parallel to its lateral plane (parallel to the bands—Figure 15b).

**Figure 16:**
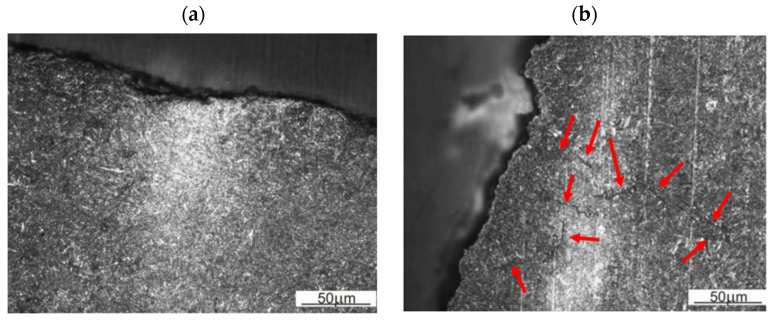
Microstructure of the forging die near the fracture surface: (**a**) fracture in the plane perpendicular to banding, (**b**) fracture in the plane parallel to banding. Red arrows indicate microcracks.

In order to assess the role of the microstructural bands in the development of the fracture, observation of the layer near the fracture surface was performed. It can be observed that the fracture approaching the band chooses it as an easier propagation path (Figure 16a). In the band near the resulting fracture, secondary microcracks can be observed, indicating the facilitation of cracking in the plastic deformation zone preceding the main crack (Figure 16b). The sites of formation of such cracks are band areas (originally inter-dendritic areas) and inter-band areas (intra-dendritic areas), where bainitic transformation occurred during quenching. The above observations indicate the important role of banding in crack development. Cracking occurs along the banding structure when the state of stress in the die does not force crack development perpendicular to the bands.

#### 3.2.6. Mechanical Properties

**Figure 17:**
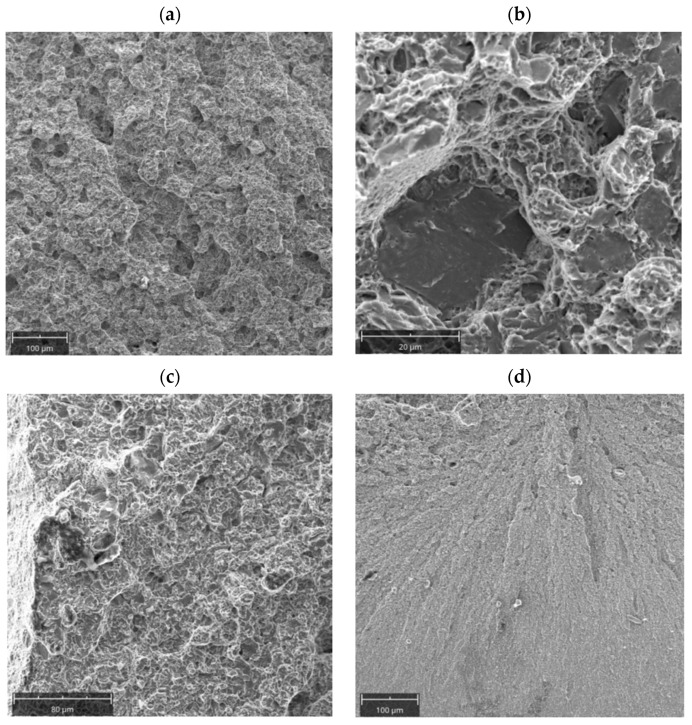
Microscopic images of the fracture surface of the Charpy sample: (**a**) quasi-brittle fracture, (**b**) brittle fracture along the grain boundaries, (**c**) area of dominating ductile fracture, (**d**) area close to the impact area of the Charpy hammer.

Charpy tests were carried out on a specimen oriented perpendicular to the banding found in the material. The impact strength was KV = 7.8 J (KCV = 9.81 J/cm^2^). In the dominant part of the surface, the fracture is quasi-brittle (Figure 17a). There are areas indicative of a brittle, trans-crystalline fracture along the planes of splitting, but in part the fracture can be described as brittle along the grain boundaries (Figure 17b). Close to the lateral edges, there is a higher proportion of ductile fracture (Figure 17c). The proportion of this zone can be described as close to 10%. This indicates that the general fracture of the Charpy sample is defined as a brittle fracture. An even different character of the fracture could be found in the area close to the impact area of the Charpy hammer (Figure 17d). Here the fracture should be considered brittle, proceeding on the splitting planes, but the extent of individual splitting areas is smaller. The die material was a brittle material, which may have contributed significantly to the resulting die failure.

In terms of mechanical testing, the hardness of the investigated material was also evaluated. The hardness was measured using the Rockwell method (C scale). The measured hardness at the level of 52 HRC indicates the application of tempering at a temperature of about 350 °C. The hardness in the near-surface zone decreased to the level of 46 HRC. This corresponds to the hardness obtained for the tested material by tempering at temperatures above 550 °C. This should be associated with the tempering of the surface layer due to the effect of heat from the forged part heated to a high temperature (about 900 °C). No increase in hardness was observed in the vicinity of the fracture, which could indicate the transformation of retained austenite into martensite during the crack development.

## 4. Conclusions

The wear mechanism of a hot working tool is a complex system of concurrent factors, which cause the tool to be inoperable prematurely. The main factors contributing to the tool failure are cracking along the mesoscopic bands (forming along former interdendritic structures) and high stresses in the deepest die cavities, resulting in fatigue cracks. Even though a vast amount of information about die manufacturing is available, the importance of intense forging of the original material still needs to be underlined.

The results presented in this paper allow us to formulate the following conclusions:

- The wear mechanism of a die (or die insert) is dependent on the location and its cavity, especially considering pronounced changes in curvature of the forged part negative shape. Fatigue cracks are formed at the points where the geometry of the die cavity changes due to material-forming operations.
- Performing heat treatment of the tool for obtaining high hardness is not a priority with regard to reducing hot working tool wear, as the initially hardened and tempered material is in almost constant contact with 1200 °C forged material. Additionally, we argue that the heat treatment reduced the overall wear resistance.
- An important wear mechanism of hot working tools is the oxidation process, occurring more rapidly at higher temperatures.
- The aerological effects of die cavity machining promote tool failure.
- Abrasive wear is characteristic of the tool surface, along which there is intense flow of the material being formed.
- The contact between the surfaces of the two dies in the case of a dynamic forging process provokes the formation of adiabatic shear bands.
- Tool cracking is associated with heat treatment, including tempering in the range of secondary hardness or tempering brittleness of the first kind. The separation of transition carbides after the boundaries of the former austenite grain is in this case unfavorable.
- The low degree of processing (low strains) of the material results in a decrease in the fracture toughness of the tool.
- The occurrence of banded microstructure results in anisotropic properties of the tool. Moreover, in the studied case the die cavities were misaligned in relation to the bands occurring in the material, being another factor in premature wear of the tool, as shown in Section 3.2.1.
- Nonmetallic inclusions, such as aluminum oxides, manganese sulfides, and molybdenum carbides, can facilitate tool fracture.

## Figures and Tables

**Table 1:** The chemical composition of the investigated tool steels.

	Chemical Composition, wt. %									
	C	Mn	Si	P	S	Cr	Mo	Ni	V	Fe
X40CrMoV5-1	0.39	0.30	0.94	0.013	0.010	4.87	1.22	0.18	0.89	Bal.
55NiCrMoV7	0.49	0.71	0.35	0.020	0.010	0.99	0.40	1.53	0.09	Bal.

## Data Availability

Not applicable.
